# Can “Googling” correct misbelief? Cognitive and affective consequences of online search

**DOI:** 10.1371/journal.pone.0256575

**Published:** 2021-09-22

**Authors:** Tetsuro Kobayashi, Fumiaki Taka, Takahisa Suzuki

**Affiliations:** 1 Department of Media and Communication, City University of Hong Kong, Hong Kong, S. A. R., China; 2 Faculty of Human Sciences, Kanagawa University, Kanagawa, Japan; 3 Department of Policy Studies, Tsuda University, Tokyo, Japan; Georgia State University, UNITED STATES

## Abstract

With increasing concern over online misinformation in perspective, this study experimentally examined the cognitive as well as the affective consequences of online search. Results of the two experiments using widely shared, prejudiced misinformation about an ethnic minority in Japan indicated that (a) online search reduces on average the likelihood of believing the misinformation, (b) the magnitude of the effect is larger among those who are predisposed to believe the misinformation, (c) cognitive correction is observed whether searchers are motivated to achieve a directional goal or an accuracy goal, and (d) online search deteriorates affective feeling toward the target groups of the misinformation. Theoretical implications are discussed in relation to the robustness of confirmation bias in online search and the “belief echo” in which exposure to negative misinformation continues to shape attitudes even after the misinformation has been effectively discredited.

## Introduction

The threat of misinformation to democratic institutions looms large. Although the scale and tangible consequences of misinformation on the electoral outcome are still being debated [1], the variety of misinformation that spread widely during the 2016 US presidential election and the 2016 United Kingdom European Union membership referendum made people realize that misinformation can lead to democratic elections becoming dysfunctional. Although the need to deal with misinformation is widely recognized, the issue is becoming increasingly urgent.

At the same time, it is becoming increasingly clear that stemming the spread of misinformation is not easy. Due to its novelty, misinformation diffuses “significantly farther, faster, deeper, and more broadly than the truth” [2, p. 1147]. As such, a broader cross-section of individuals is exposed to misinformation before corrective information reaches them, making it difficult to preemptively arrest the spread of misinformation. Furthermore, studies have indicated that it is not easy to correct misbelief once misinformation is received and accepted by individuals (for a review, see [3]). People not only tend to resist correction once they believe misinformation [4, 5], they sometimes reinforce their misbelief when presented with corrective information [6], although recent studies question the reproducibility of the backfire effect [7–9].

Misinformation is disseminated not only through social media and partisan news media, but also through interpersonal communication, making it difficult to eradicate exposure to misinformation. Therefore, it is critical whether individuals are equipped with the means of verifying the truthfulness of information when its credibility is in question. Most recent studies on misinformation and its correction have examined the effectiveness of exogenously (i.e., experimentally) provided corrective information. However, it remains unclear if and how people verify, if possible, the truthfulness of potential misinformation in real-world settings. The present study aims to fill this gap by focusing on online search, one of the most common online behaviors [10, 11].

Can online search reduce the likelihood of believing misinformation? Although online search may not be triggered when misinformation is blindly accepted, it is one of the typical actions that is taken when people are not confident of the credibility of newly encountered information. Some studies have indicated that online search can generally lead to finding credible information [12–14], while others demonstrate that it does not necessarily lead to truthful information due to the well-documented confirmation bias [15–18]. That is, the predispositions of online search users can powerfully direct their search behavior, and the resulting selectivity in the exposure to and acceptance of search results can strengthen, rather than correct, misbelief [19, 20]. Given this background, whether online search can reduce the likelihood of believing misinformation or not is a crucial empirical question.

## Epistemic uncertainty and online search

Although individuals may have numerous goals when using search engines [21], one of the primary goals of online search is to reduce epistemic uncertainty. When confronted with information that elicits uncertainty, people face the need to impose an adequate structure on the perception of their social environment, leading to the need for orientation [22]. While traditional mass media played an essential role in meeting the need for orientation in the pre-Internet era, online search is now an indispensable means for reducing epistemic uncertainty. The growing demand for online search to meet the need for orientation has resulted in the “Googlization of our lives” [23], wherein people rely heavily on search engines to sift out credible information on economic, health, social, and political aspects of life. Online search is particularly important in the political domain because the extant literature consistently indicates that political events, news, and campaigns trigger online search [14, 24].

Online search is particularly relevant to verifying the authenticity of political information. Because online searchers seek information that they do not yet possess, online search has significant potential to update preexisting beliefs [25, 26]. Therefore, to the extent that search engines are capable of providing accurate information, they are expected to arrest the spread of misinformation and consequential misbelief. In particular, when people encounter potential misinformation and turn to the Internet for fact-checking, search engines are expected to provide credible information and prevent people from believing political misinformation. However, there is ample evidence that online search does not necessarily lead to credible information and arrest of misinformation. The primary mechanism that prevents online searchers from reaching truthful information is confirmation bias arising from motivated reasoning and selective exposure.

## Confirmation bias in online search

Although people can generally gain credible information using search engines [12–14], studies have strongly suggested that motivated reasoning and selective exposure in online search can lead to confirmation bias even when people search for correct, reliable information. That is, rather than reaching out to objectively accurate information, confirmation bias in online search can lead searchers to pay selective attention to, and accept, the information that reinforces their preexisting attitudes [15–18]. As a result, especially for those who are predisposed to believe particular misinformation, online search can end up with users gaining even less accurate information, resulting in reinforcement of misbelief.

Predispositions relevant to the topic of search powerfully shape the consequences of search in a variety of ways. First, online searchers are known to browse only a limited number of search results, typically the first few presented on the first page [27]. When selecting the outcomes of the search results to browse, online searchers tend to engage in selective exposure whereby the search results that are congruent with the predispositions are more likely to be selected than the incongruent results. Second, online searchers do not necessarily interpret and accept the search results that they choose to browse in a fair and neutral way. Well-documented motivated reasoning suggests that even exposure to the same information can elicit remarkably divergent interpretations depending on an individual user’s predispositions [16, 28, 29]. Third, search engines learn a user’s predispositions from the search history as well as from the records from other affiliated services. They continuously update the algorithms that personalize the search results so that they become more congruent with the user’s predispositions. Therefore, algorithmic personalization of online search engines tends to work to reinforce, rather than alter or correct, preexisting attitudes [30, 31].

All these mechanisms facilitate confirmation bias in online search. Indeed, there is ample evidence that demonstrates confirmation bias in online search (e.g., [16, 17, 19]). As an illustration, by analyzing participants’ beliefs regarding a medical issue before and after reviewing online search results, White [32, p. 2165–2166] finds that “presearch beliefs are affected only slightly by searching” and “presearch beliefs affect search behavior,” leading to the conclusion that “search engines offer little assistance in helping searchers form factually correct beliefs.”

Therefore, to the extent that online search is colored by confirmation bias, online search will increase the likelihood of believing misinformation among those who are predisposed to believe misinformation. However, for the same reason, online search will reduce the likelihood of believing the misinformation among those who are predisposed to *disbelieve* it because they are more likely to browse the search results that debunk the misinformation and discredit the search results that support the misinformation. We therefore formulate the following two hypotheses.

H1: Online search increases the likelihood of believing misinformation among those who are predisposed to believe misinformation.

H2: Online search reduces the likelihood of believing misinformation among those who are predisposed to disbelieve misinformation.

## Belief echo in online search

To the extent that it leads to cognitive correction of misbelief, online search is a useful tool for fact-checking. However, previous studies have indicated that cognitive correction of misinformation does not necessarily lead to an improvement in the affective evaluation of the target of misinformation [3, 5, 33]. The notion that is particularly relevant as an affective consequence of online search is “belief echo” [34], which suggests that cognitive correction as a result of online search does not necessarily ameliorate the feeling toward the groups that are negatively portrayed in misinformation.

Thorson [34] argues that belief echo stems from the two processes of automatic updating of online tallies and conscious reasoning. First, the automatic process is driven by the asymmetric affective responses to misinformation and correcting information. All else being equal, exposure to negative information against groups or individuals invokes stronger affective responses than does exposure to fact-based correcting information. Because online tallies are more effectively updated by stronger affective responses, the negative shift of the online tally produced by the initial exposure to misinformation tends to be larger than the subsequent positive pushback by the correction. Therefore, when misinformation includes negative information about specific targets, the initial negative updating of the online tallies attached to the targets cannot be fully undone by cognitive correction.

Second, the conscious process of belief echo is driven by the “no smoke without fire” type of conscious reasoning, whereby individuals recognize that a particular piece of information is objectively false but infer that the existence of misinformation suggests the likelihood of other negative information being true. Due to this line of conscious reasoning, the negative impact of the initial exposure to the misinformation on the evaluation of the politician cannot be removed by correction [35]. As a result of these automatic and conscious processes, “exposure to a piece of misinformation can shape a person’s attitudes despite the fact that she recognizes it is false” [34, p. 461].

When seeking the truthfulness of negative misinformation online, searchers can be exposed to a large amount of negative information about the target of the misinformation, even though they may eventually conclude that the information is false. Even the credible websites prioritized in search results tend to refer to specific misinformation as the target of criticism, which, as a by-product, facilitates the exposure to misinformation. Therefore, drawing upon the process of belief echo, we propose the following hypothesis.

H3: Online search deteriorates the overall feeling toward the target of misinformation.

## The case of the Zainichi Koreans in Japan

To investigate the cognitive and affective consequences of online search, we use the case of misinformation concerning Korean citizens who have residency status in Japan (hereafter, Zainichi Koreans). Zainichi Koreans are long-term Korean residents in Japan who trace their roots back to Korea under Japanese colonial rule. Discrimination and hate speech against Zainichi Koreans loom large both offline and online, which in August 2014 led the UN Committee on the Elimination of Racial Discrimination (CERD) to call on Japan to address more firmly the hate speech and racism directed against Zainichi Koreans. In doing so, CERD expressed specific concern about the hate speech based on misinformation spread online and called for appropriate measurements to tackle this issue.

We use the specific misinformation that states “More than 20% of those who are on welfare are Zainichi Koreans.” According to the national surveys on social welfare recipients conducted by the Ministry of Health, Labour, and Welfare in 2015, the total number of welfare recipients was 2,127,841, of whom 37,139 were from households where the head was of Korean nationality [36]. Therefore, the true figure for Zainichi Koreans on welfare is only about 1.7%; thus, the statement “More than 20%” is objectively false. Nevertheless, this misinformation is widely believed among the Japanese public, as will be shown in the following analyses.

## Study 1

The entire experiment was fielded online. First, Japanese adults aged 20–59 years were recruited from the online panel of a leading online survey firm in Japan on October 16, 2015 (N = 1,032). After measuring pretreatment covariates, the participants were randomly assigned to either the treatment or the control group (n = 516 for each group) and then invited to the online search experiment on October 30, 2015. The participants were instructed to search online until they became confident that the given statement was true or false. They searched for a maximum of 10 min using any search engine. To ensure that the participants fully engaged in online search, they were not allowed to proceed to the measurement of outcomes until 5 min had passed. Those who spent 10 min were automatically redirected to the measurement of outcomes. Before the participants started to search, they were instructed that they would be asked to copy and paste the URL of “the most informative website during the search” for making the final judgment of authenticity. The participants were then requested to open their search in a different window or tab from the main survey site. Once the participants reached a conclusion with confidence, they copied and pasted the URL of “the most informative website during the search” and responded to posttreatment outcomes. The number of participants who completed the entire experiment was 257 for the treatment group and 253 for the control group.

### Treatment and measurement in Study 1

#### Treatment

The treatment in this experiment is online search about the truthfulness of the misinformation about Zainichi Koreans. The treatment group was instructed to search to judge if the statement “More than 20% of those who are on welfare are Zainichi Koreans” is “objectively true or false.” The control group searched regarding the truthfulness of the placebo statement “Less than 10% of those who shopped online in 2012 encountered some trouble in their transactions.” A survey by the Ministry of Economy, Trade and Industry [37] indicated that the ratio was about 30%, rendering this placebo statement false.

#### Pretreatment covariates

Sex (Male: 61.76%), age (*M* = 40.24, *SD* = 11.12), education (1: less than college = 24.51%, 2: some college = 23.53%, 3: bachelor’s degree or above = 51.96%), and party identification (Liberal Democratic Party (LDP) = 30.39%, Democratic Party of Japan (DPJ) = 7.45%, Japan Restoration Party (JRP) = 3.14%, Japanese Communist Party (JCP) = 2.94%) were measured. Party identification was included as a covariate because politicians from conservative parties, such as the JRP, tend to problematize the allegedly high percentage of Zainichi Koreans among those on welfare [38, 39].

To test H1 and H2, the feeling thermometer score of South Korea (as a country) was measured as the predisposition to believe the misinformation about Zainichi Koreans. The score was measured on an 11-point scale and rescaled to range from 0 to 1 (*M* = 0.24, *SD* = 0.23). By analyzing the xenophobic discourse against Zainichi Koreans in Japan, Higuchi [40] demonstrates that the discrimination against Zainichi Koreans is increasingly associated with the international conflicts between Japan and South Korea. That is, the escalation of conflicts between Japan and South Korea over historical and territorial issues, such as the comfort women in World War II, is intensifying negative feeling toward Korean people in general; thus, those Japanese who dislike South Korea tend to have negative feeling toward Zainichi Koreans as its domestic surrogates. It is, therefore, reasonable to assume that those who have negative feeling toward South Korea are predisposed to believe the negative misinformation about Zainichi Koreans.

#### Posttreatment outcomes

To test H1 and H2, the subjective truth of the statement “More than 20% of those who are on welfare are Zainichi Koreans” was dichotomously measured (1: True = 31.92%, 0: False = 68.08%). To test H3, feeling thermometer scores of South Koreans and Zainichi Koreans were measured using an 11-point scale and rescaled to range from 0 to 1 (South Koreans: *M* = 0.30, *SD* = 0.25; Zainichi Koreans: *M* = 0.35, *SD* = 0.24).

### Results of Study 1

Approximately the same number of participants used Google (47.06%) or Yahoo! Japan (46.47%) for their search. In the following analyses, however, we did not find any differences depending on the search engine used. Despite the relatively large attrition, covariate balance was maintained (S1 File).

**Fig 1:**
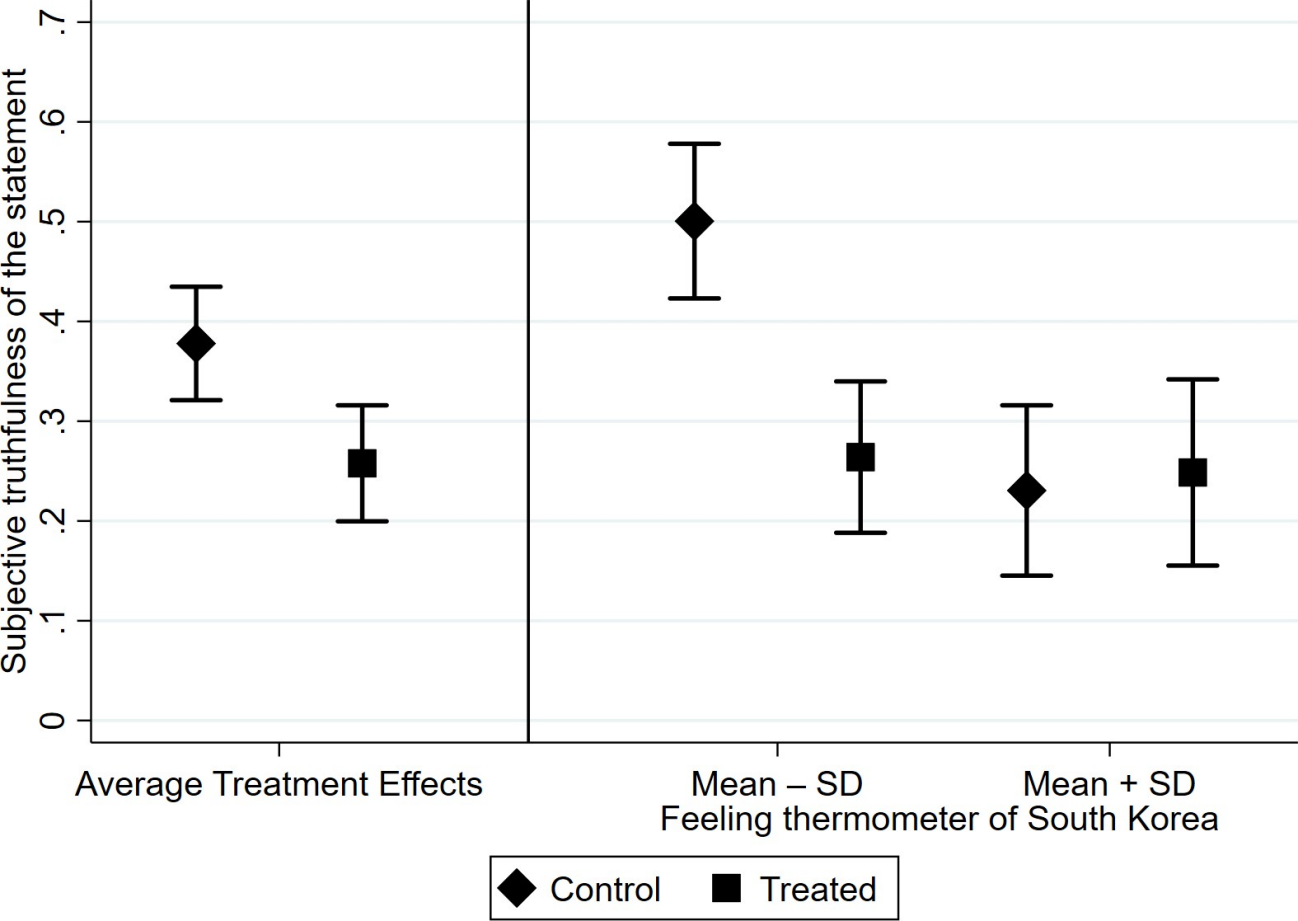
Average treatment effects and the interaction between the treatment and feeling thermometer score of South Korea (error bars are 95% confidence intervals).

We begin by testing the average treatment effect of online search. Although it was not explicitly hypothesized due to the expected heterogeneity of the treatment effects (i.e., H1 and H2), it is crucial to know whether online search increases or decreases the likelihood of believing misinformation on average. Two ordinary least squares (OLS) regression models were developed with subjective truthfulness of the statement as the dependent variable and the treatment as the independent variable. In Table 1, Model 1 was estimated without pretreatment covariates, while Model 2 was estimated with covariates with the aim of increasing the efficiency of estimation. The two models consistently show statistically significant negative average treatment effects on subjective truthfulness of the statement, which indicates that online search reduces on average the likelihood of believing the misinformation. This average treatment effect is illustrated in the left panel of Fig 1. While 37.94% of the control group believed that the misinformation was true, it fell to 25.62% in the treatment group. It is also important to note that more than one-quarter of the treatment group still believed the misinformation to be true, suggesting the prevalence of this misinformation among the Japanese public.

**Table 1:** Estimation of treatment effects (Study 1).

	Subjective truthfulness of the misinformation				Feeling thermometer of the South Koreans		Feeling thermometer of the Zainichi Koreans	
	Model 1	Model 2	Model 3	Model 4	Model 5	Model 6	Model 7	Model 8
	Coef. (B)							
Treatment	–0.123**	–0.120**	–0.256**	–0.258**	–0.055*	–0.061**	–0.032	–0.041*
	(0.042)	(0.042)	(0.060)	(0.060)	(0.022)	(0.021)	(0.021)	(0.021)
Feeling thermometer score of South Korea			–0.593**	–0.565**				
			(0.123)	(0.126)				
Treatment			0.509**	0.533**				
× Feeling thermometer score of South Korea			(0.179)	(0.179)				
Constant	0.379**	0.599**	0.531**	0.772**	0.330**	0.237**	0.370**	0.207**
	(0.029)	(0.107)	(0.043)	(0.112)	(0.015)	(0.054)	(0.015)	(0.053)
Covariates	No	Yes	No	Yes	No	Yes	No	Yes
Observations	495	495	495	495	510	510	510	510
R–squared	0.017	0.048	0.062	0.086	0.013	0.081	0.005	0.091

Standard errors in parentheses. Covariates include sex, age, education, and party identities (LDP, DPJ, JRP, and JCP).

** *p* < 0.01

* *p* < 0.05

+ *p* < 0.1.

Next, To test H1 and H2, OLS regression models were estimated with subjective truthfulness of the statement as the dependent variable and the treatment and its interaction with the feeling thermometer score of South Korea as the independent variable (Model 3 without covariates and Model 4 with covariates). As shown in Model 3 and 4 in Table 1, the interaction term consistently showed a statistically significant positive effect. For a more intuitive understanding of the interaction effect, the right panel of Fig 1 presents the predicted values of the subjective truthfulness of the statement based on Model 4 for those who were predisposed to believe the misinformation (feeling thermometer score of South Korea = Mean–SD) and those who were predisposed to disbelieve the misinformation (feeling thermometer score of South Korea = Mean + SD).

Those respondents with a lower feeling thermometer score of South Korea in the control group showed a higher likelihood of believing the misinformation (50.06%) than the control group of the entire sample (37.94%), indicating that the feeling thermometer of South Korea taps the predisposition to believe the misinformation, as expected.

Among those who are predisposed to believe the misinformation; i.e., those with a lower feeling thermometer score of South Korea, the treatment group showed significantly lower belief of the subjective truth of the statement (26.40%) compared with their counterparts in the control group (50.06%). This result not only rejects H1—it reflects the opposite of H1. That is, online search *reduces* the likelihood of believing misinformation among those who are predisposed to believe the misinformation.

On the other hand, among those who are predisposed to disbelieve the misinformation, i.e., those with a higher feeling thermometer score of South Korea, the subjective truth of the statement for the treatment group (24.86%) was not statistically distinguishable from that of the control group (23.06%). That is, online search did not reduce the likelihood of believing misinformation among those who were predisposed to disbelieve the misinformation, which does not support H2. Because more than one-quarter of the treatment group still believed the misinformation to be true, it is difficult to interpret this result as a floor effect, given that there was significant room for cognitive correction.

Next, to test H3, OLS regression models were estimated with posttreatment feeling thermometer scores of South Koreans and Zainichi Koreans as the dependent variables (Models 5–8 in Table 1). Models 5 and 7 were estimated without covariates, while Models 6 and 8 were estimated with covariates. The interaction term was not included because online searchers are expected to be exposed to the negative information about the target groups regardless of their predispositions.

Models 5 and 6 indicate that, regardless of the inclusion of covariates, online search reduces the feeling thermometer scores of South Koreans by approximately 0.06, which is statistically significant. This pattern was largely consistent when the dependent variable was the feeling thermometer of Zainichi Koreans (Models 7 and 8). Although the treatment effect did not reach the conventional level of statistical significance in Model 7, it did so when covariates were included (Model 8). On average, online search lowered the feeling thermometer scores toward Zainichi Koreans by 0.03–0.04. These results support H3. That is, although online search reduces the likelihood of believing the misinformation, especially among those who are predisposed to believe it, it also deteriorates the affective feeling toward the target groups of the misinformation.

To probe into the observed pattern of effects, we accessed and saved all the contents of websites reported by participants as “the most informative website during the search,” which was subsequently used for content analysis. We summarize the key findings here, and present the full results in S2 File, including the coding rules and intercoder reliabilities. The results of content analysis did not provide solid evidence of confirmation bias. Even among those who were predisposed to believe the misinformation, the majority of the websites they found most informative were fact-based, neutral websites, which arguably led to the reduction of misbelief. However, it was also seen that the websites with a negative tone toward Zainichi Koreans were more likely to be considered as informative than those with a positive tone, suggesting that the available information was lopsidedly negative. As negative affective response invoked by initial exposure to negative information leads to updating of online tallies, it is expected to have a persistent affective effect even after the misinformation is corrected, resulting in belief echo.

### Discussion of Study 1

Study 1 indicated that online search reduces, on average, the likelihood of believing misinformation. This effect was heterogeneous; online search reduces the likelihood of believing misinformation among those who are predisposed to believe the misinformation, but not among those who are predisposed to disbelieve the misinformation. Despite the successful cognitive correction, online search showed detectable negative effects on the overall feeling toward South Koreans and Zainichi Koreans, which corroborates the argument of belief echo.

Although the tests of H1 and H2 did not support confirmation bias in online search, interpretation of the results is left with ambiguity because the goals of search were not exogenously manipulated. Confirmation bias is expected to come into play when searchers seek information that reinforces their predispositions rather than objectively credible information. In this respect, the participants in Study 1 were instructed to search to judge if the statement was “objectively true or false,” but this reference to objectivity could have motivated the participants to seek objectively credible information, resulting in suppression of confirmation bias.

The two goals that are most relevant to online search are *accuracy* and *directional* goals. An accuracy goal is defined as “the desire to hold attitudes and beliefs that are objectively valid” [41, p. 556], which results in balanced information seeking and extensive elaboration of the available information [42]. When people are motivated to pursue an accuracy goal, they seek information with the aim of forming a preference that is “a correct or otherwise best conclusion” [29, p. 756]. Although individuals with strong prior attitudes tend to engage in motivated reasoning, they still endeavor to achieve an accuracy goal when, for instance, they are high in need for cognition [43] or they are incentivized to be accurate by material benefits [44], resulting in even-handed and unbiased seeking of information regardless of their preexisting attitudes [45].

In contrast, when people are motivated to achieve a directional goal, they tend to seek information with the aim of protecting self-relevant attitudes, which results in “deeper and more favorable elaboration of arguments supporting those attitudes than arguments opposing them” [42, p. 715]. By selectively heeding the information that corroborates preexisting attitudes, people motivated to achieve a directional goal tend to resist updating, sometimes even reinforcing their attitudes [29, 46]. A directional goal also triggers motivated reasoning through which individuals interpret newly obtained information in a biased way, so that they can avoid cognitive dissonance with their extant attitudes [47]. As a result, when motivated to pursue a directional goal, individuals are not only unlikely to scrutinize relevant information in an unbiased way, but they are also likely to misinterpret the information that might otherwise be useful in reaching an accurate conclusion [48, 49].

It is, therefore, expected that online search increases the likelihood of believing misinformation among those who are predisposed to believe misinformation (H1), *especially when they engage in search with a directional goal*. In a similar vein, online search will reduce the likelihood of believing misinformation among those who are predisposed to disbelieve misinformation (H2), *especially when they engage in search with a directional goal*. In contrast, when searchers are motivated to achieve an accuracy goal, the likelihood of believing misinformation will be reduced, regardless of their predispositions. To test these more nuanced versions of H1 and H2, Study 2 was designed which experimentally manipulates the goals of online search.

## Study 2

Japanese adults aged 20–59 years were recruited from a leading online crowdsourcing platform in Japan on January 31, 2017 (N = 2,189). The design of the experiment was identical to Study 1, except in two points. First, the participants were instructed to search online immediately after responding to the pretreatment covariates with the aim of minimizing the attrition between the pretreatment measurement and the search. Second, three treatment groups instead of one were set up to test the effects of goals. One of the three treatment groups was the same as the treatment group in Study 1 (hereafter, the original treatment group). The other two were the directional goal group and the accuracy goal group. The number of participants who completed the entire experiment was 546 for the original treatment group, 548 for the directional goal group, 543 for the accuracy goal group, and 552 for the control group.

### Treatment and measurement in Study 2

#### Treatment

Study 2 used the same two statements that were used in Study 1: “More than 20% of those who are on welfare are Zainichi Koreans” and “Less than 10% of those who shopped online in 2012 encountered some trouble in their transactions.” The instructions for the original treatment group and the control group were identical to those in Study 1.

To experimentally manipulate the goals of the search, the conventional psychological approaches to induce accuracy and directional motivation (e.g., [28, 50]) were adjusted to the context of online search (see [45] for a similar approach). Specifically, the directional goal group was instructed to “search for information that is as agreeable as possible.” In contrast, the accuracy goal group was instructed to “search for information that is as objectively accurate as possible, regardless of your own opinion.”

#### Pretreatment covariates

Sex (Male: 47.88%), age (*M* = 35.40, *SD* = 9.55), education (1: less than college = 19.42%, 2: some college = 23.30%, 3: bachelor’s degree or above = 57.29%), and party identification (LDP = 43.49%, DPJ = 6.26%, JRP = 5.25%, JCP = 4.84%) were measured. The feeling thermometer score of South Korea (i.e., the predisposition to believe the misinformation) was measured on an 11-point scale and rescaled to range from 0 to 1 (*M* = 0.24, *SD* = 0.20).

To facilitate the interpretation of content analysis, we also measured the pretreatment subjective truthfulness of the statement from the three treatment groups (i.e., the lagged dependent variable). That is, before they searched online, participants responded if they believed the statement “More than 20% of those who are on welfare are Zainichi Koreans” was true or not (1: True = 40.68%; 0: False = 59.32%). This question was not asked for the control group so as not to encourage the participants in the control group to search about Zainichi Korean welfare recipients, which would undermine the internal validity. Therefore, this item was not used for the estimation of treatment effects and was only used for the content analysis of the websites that were found to be most informative.

#### Posttreatment outcomes

Subjective truthfulness of the statement “More than 20% of those who are on welfare are Zainichi Koreans” was dichotomously measured (1: True = 34.72%; 0: False = 65.28%). The feeling thermometer scores of South Koreans and Zainichi Koreans were measured on an 11-point scale and rescaled to range from 0 to 1 (South Koreans: *M* = 0.34, *SD* = 0.23; Zainichi Koreans: *M* = 0.37, *SD* = 0.23).

### Results of Study 2

The number of participants who used Google (62.95%) was double those who used Yahoo! Japan (31.29%). In the following analyses, however, we did not find any differences depending on the search engine used. Covariate balance was maintained (S3 File).

**Fig 2:**
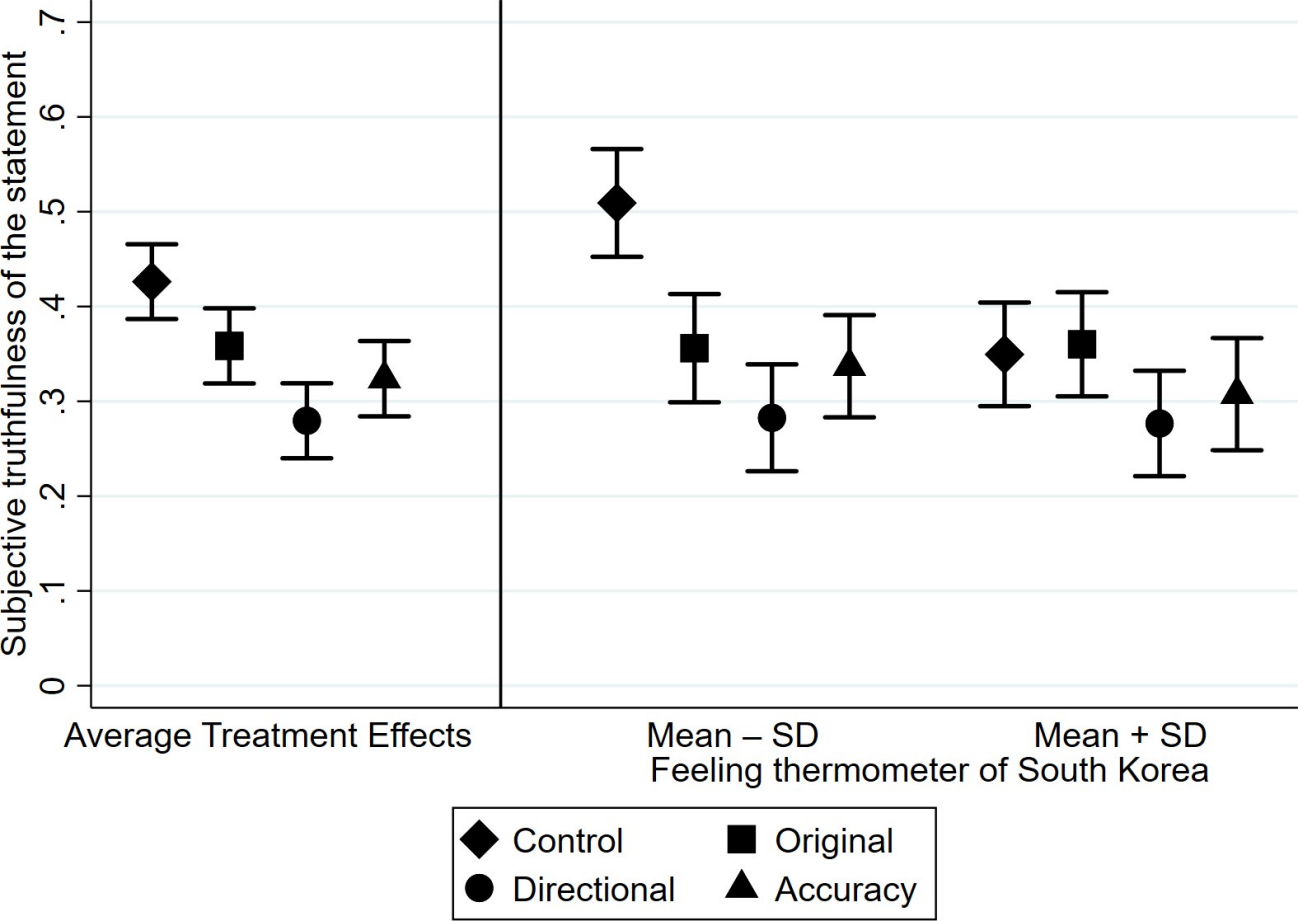
Average treatment effects and the interaction between the treatments and the feeling thermometer score of South Korea (error bars are 95% confidence intervals).

As in Study 1, we begin by estimating the average treatment effects. OLS regression models were estimated with subjective truthfulness of the statement as the dependent variable and the three types of treatment as the independent variables (Models 1–4 in Table 2). Model 1 in Table 2 was estimated without pretreatment covariates, while Model 2 was estimated with covariates. Results indicate that, regardless of the inclusion of covariates, the original treatment consistently showed a statistically significant negative effect on subjective truthfulness of the statement, thereby replicating Study 1. It is also notable that both directional goal and accuracy goal groups showed a significant reduction in misbelief compared with the control group. These results strongly indicate that online search on average reduces the likelihood of believing misinformation. These average treatment effects are illustrated in the left panel of Fig 2.

**Table 2:** Estimation of treatment effects (Study 2).

	Subjective truthfulness of the misinformation				Feeling thermometer of the South Koreans		Feeling thermometer of the Zainichi Koreans	
	(0/1)							
	Model 1	Model 2	Model 3	Model 4	Model 5	Model 6	Model 7	Model 8
	Coef. (B)							
Original treatment	–0.069*	–0.068*	–0.170**	–0.172**	–0.021	–0.027*	–0.023	–0.027*
	(0.029)	(0.029)	(0.046)	(0.046)	(0.014)	(0.013)	(0.014)	(0.014)
Directional goal treatment	–0.147**	–0.147**	–0.236**	–0.245**	–0.022	–0.026+	–0.032*	–0.035**
	(0.029)	(0.028)	(0.046)	(0.046)	(0.014)	(0.013)	(0.014)	(0.013)
Accuracy goal treatment	–0.100**	–0.102**	–0.184**	–0.187**	–0.027+	–0.033*	–0.042**	–0.046**
	(0.029)	(0.029)	(0.045)	(0.045)	(0.014)	(0.014)	(0.014)	(0.014)
Feeling thermometer score of South Korea			–0.393**	–0.406**				
			(0.102)	(0.102)				
Original treatment			0.409**	0.417**				
× Feeling thermometer score of South Korea			(0.145)	(0.144)				
Directional goal treatment			0.357*	0.391**				
× Feeling thermometer score of South Korea			(0.145)	(0.145)				
Accuracy goal treatment			0.332*	0.331*				
× Feeling thermometer score of South Korea			(0.146)	(0.145)				
Constant	0.426**	0.535**	0.524**	0.645**	0.360**	0.410**	0.396**	0.357**
	(0.020)	(0.051)	(0.032)	(0.058)	(0.010)	(0.024)	(0.010)	(0.024)
Covariates	No	Yes	No	Yes	No	Yes	No	Yes
Observations	2,189	2,189	2,189	2,189	2,189	2,189	2,189	2,189
R–squared	0.013	0.023	0.019	0.030	0.002	0.053	0.005	0.049

Standard errors in parentheses. Covariates include sex, age, education, and party identities (LDP, DPJ, JRP, and JCP).

** *p* < 0.01

* *p* < 0.05

+ *p* < 0.1.

Next, we included the interaction term between the treatments and the feeling thermometer scores of South Korea (Models 3 and 4 in Table 2). Model 3 was estimated without covariates, while Model 4 was estimated with covariates. All the interaction terms showed significant effects regardless of the inclusion of covariates. To put these interaction effects in context, the right panel of Fig 2 presents the predicted values of the subjective truthfulness of the statement based on Model 4 for those who were predisposed to believe the misinformation (feeling thermometer score of South Korea = Mean–SD) and those who were predisposed to disbelieve the misinformation (feeling thermometer score of South Korea = Mean + SD).

The original treatment significantly reduced the likelihood of believing the misinformation among those who were predisposed to believe it (i.e., those who had a lower feeling thermometer score of South Korea), but did not among those who were predisposed to disbelieve it, which replicates the results of Study 1. More importantly, among those who were predisposed to believe the misinformation, the directional goal treatment showed a *negative* rather than a positive effect on the likelihood of misbelief, which again is at odds with confirmation bias. In other words, those who were predisposed to believe the misinformation corrected their misbelief, even when they were instructed to search for information that was agreeable to their prior opinion. In fact, the reduction of misbelief in the directional goal group was even larger than that in the original treatment group. Once again, these results reject H1.

The accuracy goal treatment showed a significant negative effect among those who were predisposed to believe the misinformation, as expected. In contrast, the directional goal, as well as the accuracy goal, treatment did not show any detectable effect on the likelihood to believe the misinformation in those who were predisposed to disbelieve the misinformation, which again rejects H2. In summary, the pattern of observed effects indicates that online search reduces misbelief regardless of the searchers’ goals.

Next, to test H3, OLS regression models were estimated with posttreatment feeling thermometer scores of South Koreans and Zainichi Koreans as the dependent variables (Models 5–8 in Table 2). Models 5 and 7 were estimated without covariates, while Models 6 and 8 were estimated with covariates. Although the treatment effects did not reach a conventional level of statistical significance in Model 5, the original treatment group and the accuracy goal group showed a more negative feeling than the control group when covariates were included (Model 6). Similarly, all three treatment groups showed statistically significant negative effects on feeling thermometer scores of Zainichi Koreans (Model 8), replicating the finding of Study 1. In summary, regardless of the goals of search, seeking information regarding the misinformation led to a more negative feeling toward the target groups of the misinformation, despite the fact that participants became less likely to believe the misinformation to be true. These results support H3 and the argument of belief echo.

As in Study 1, we accessed and saved all the contents of websites reported by the participants as “the most informative website during the search,” which was subsequently used for content analysis. We summarize the key findings here and present the full results in S4 File, including the coding rules and intercoder reliabilities. Combined with the pretreatment subjective truthfulness of the statement, content analyses of the websites provided two major insights. First, like Study 1, confirmation bias was weak at best and most participants found fact-based, neutral websites to be the most informative, which arguably led to the overall corrective effect of online search. At the same time, heavier reliance on the websites with a negative tone toward Zainichi Koreans than those with a positive tone was indicated to be the source of belief echo. Second, those who found neutral or positive websites to be informative were more likely to correct the misbelief than to newly believe the misinformation, while those who found negative websites to be informative were more likely to newly believe the misinformation than to correct the misbelief. These results suggest that online search can aggravate the misbelief, especially when searchers perceive the information value of websites with a negative tone toward the target groups.

### Discussion of Study 2

Study 2 exogenously manipulated the goals of online search to see if those who are predisposed to believe the misinformation show confirmation bias when they are directed to confirm their predisposition, and if those who are predisposed to disbelieve the misinformation show significant reduction of misbelief. The results indicated that this was not the case: that is, online search reduces misbelief on average regardless of the goals. In particular, it does not support the well-documented confirmation bias in online search because the largest reduction of misbelief was observed when a directional goal was induced among those who were predisposed to believe the misinformation. These results suggest that online search has a robust cognitive effect to alleviate misbelief, regardless of the motivation of the search. Study 2 also replicated the negative effect of online search on the affective feeling toward South Korea and Zainichi Koreans, which is in marked contrast to the reduction of misbelief.

## General discussion

With the increasing concern over online misinformation and disinformation, this study experimentally examined the cognitive, as well as the affective, consequences of online search. The consistent and robust average treatment effects to reduce the likelihood of believing the misinformation suggest that confirmation bias in online search may not be as robust as has been documented. While Nyhan and Reifler [6] reported a “backfire effect” by demonstrating that debunking a claim can make those who are predisposed to believe the claim end up believing it more strongly rather than less, recent studies have failed to replicate the effect [7–9]. Through five experiments with more than 10,000 participants, [9, p. 135] conclude that “citizens heed factual information, even when such information challenges their ideological commitments.” Our findings are consistent with this conclusion; those predisposed to believe the misinformation can effectively correct their misbelief by searching for credible information online. After all, “[m]otivation can color our judgments, but we are not at liberty to conclude whatever we want to conclude simply because we want to” [51, p. 224]. That said, although search is a common online behavior, the scale of corrective effect of online search in the real world setting has yet to be clarified because those who are strongly predisposed to believe misinformation may not bother to search and check the truthfulness of the information. The findings of the study should be carefully interpreted by taking into account the frequency of online search when people encounter potential misinformation in nonexperimental, real-world settings.

The other consistent finding that online search deteriorates affective evaluation of the target groups of misinformation is in line with the argument of belief echo [34]. That is, given the substantial amount of negative misinformation about Zainichi Korean welfare recipients available online, exposure to this information at the early stage of search invokes negative affect, leading to negatively updating the online tallies attached to the target groups of the misinformation. As correction or fact-based information produces weaker affective pushback, the negative updating of online tallies cannot be fully rectified even when searchers find out that the information is false. It is a serious side effect of fact-checking through online search because lowered feelings toward the target group predispose searchers to believe other misinformation about the groups in the future. That is, even when specific information is disconfirmed as a result of online search, that search can facilitate other types of misbelief due to the deteriorated feeling toward the groups. This recursive mechanism arguably makes it difficult to eliminate misbelief even when there is ample correction of information available online.

Several limitations remain to be addressed. First, while the participants in this study were instructed to search for misinformation concerning Zainichi Koreans, it is not clear whether the same results would be obtained in different contexts. Cross-cultural studies are called for to examine the external validity of the findings in different cultural contexts. Second, the manipulation of goals in Study 2 has room for improvement. In particular, no established method to prime a directional goal has been reported [45], although an accuracy goal is known to be more effectively manipulated [28, 45, 50]. Future studies need to develop the methods to reliably manipulate different types of goals. Third, although the content analyses of the most informative websites partially unpacked the process through which online search has cognitive as well as affective consequences, this study was not able to track all the pages that the participants browsed during the search. To isolate the information that is critical for the likelihood of believing or disbelieving misinformation, future studies should digitally track the entire browsing behavior during online search.

## Supporting information

S1 File Covariate balance of Study 1. (DOCX)

S2 File Content analysis of the most informative websites (Study 1). (DOCX)

S3 File Covariate balance of Study 2. (DOCX)

S4 File Content analysis of the most informative websites (Study 2). (DOCX)
