# Supplementary material for: Can “Googling” correct misbelief? Cognitive and affective consequences of online search
Source: PLoS One. 2021 Sep 22;16(9):e0256575. doi: 10.1371/journal.pone.0256575 (PMC8457483; doi:10.1371/journal.pone.0256575)
Supplement: S1 File — (DOCX) [file pone.0256575.s001.docx]

**S1 File. Covariate balance of Study 1**

|  | Control | Treatment | Chi-squared test | *t* test |
| --- | --- | --- | --- | --- |
| % Female | 39.53 | 36.96 | χ^2^(1) = 0.35, p = 0.55 |  |
| Age | 39.50 | 40.97 |  | *t*(508) = –1.50, *p* = 0.14 |
| Education (1–3) | 2.28 | 2.27 |  | *t*(508) = 0.16, *p* = 0.87 |
| % Party identity: LDP | 31.62 | 29.18 | χ^2^(1) = 0.36, p = 0.55 |  |
| % Party identity: DPJ | 8.30 | 6.61 | χ^2^(1) = 0.53, p = 0.47 |  |
| % Party identity: JRP | 3.95 | 2.33 | χ^2^(1) = 1.10, p = 0.30 |  |
| % Party identity: JCP | 3.16 | 2.72 | χ^2^(1) = 0.09, p = 0.77 |  |
