# Supplementary material for: Can “Googling” correct misbelief? Cognitive and affective consequences of online search
Source: PLoS One. 2021 Sep 22;16(9):e0256575. doi: 10.1371/journal.pone.0256575 (PMC8457483; doi:10.1371/journal.pone.0256575)
Supplement: S2 File — (DOCX) [file pone.0256575.s002.docx]

**S2 File. Content analysis of the most informative websites (Study 1)**

We accessed and saved all the contents of websites reported by the participants as “the most informative website during the search.” Two trained coders independently coded the contents of those websites. There were 44 unique websites among the 203 responses. The coding indicated that 33.00% of the 203 responses were blogs, followed by online news websites (24.14%), and websites of traditional mass media (12.32%). Cohen’s Kappa was 0.56.

After excluding 11 unique websites that were irrelevant to the issue of Zainichi Korean welfare recipients (20 responses), the coders categorized the remaining 183 responses into one of three types. 1: Websites that explicitly accuse Zainichi Koreans of illegitimately receiving welfare (32.24%; hereafter, negative websites); 2: websites that explicitly defend the Zainichi Koreans for legitimately receiving welfare (9.29%; hereafter, positive websites); and 3: websites that are fact-based and neutral regarding Zainichi Korean welfare recipients (58.47%; hereafter, neutral websites). Cohen’s Kappa was 0.58.

To examine how participants searched differently depending on their predisposition to believe misinformation, the distribution of the three categories of “the most informative website during the search” is illustrated for the different levels of the feeling thermometer of South Korea (Figure OA1). Note that Figure OA1 only uses the data from the treatment group because we are interested in online search about the misinformation regarding Zainichi Koreans. The proportion of negative websites showed an indistinct negative correlation with the feeling thermometer score of South Korea of 27.50%, 32.26%, and 34.57% for the high, middle, and low feeling thermometer groups, respectively. Although this pattern is consistent with confirmation bias, it was not a strong relationship and the chi-squared test did not reject the independence between the feeling thermometer and the types of the most informative websites (χ^2^(4) = 1.17, *p* = 0.88). It should also be noted that, even among those with low feeling toward South Korea, more than half (58.02%) of the searchers indicated that fact-based, neutral websites were the most informative. These neutral websites were more likely than the other two types of websites to provide official statistics about Zainichi Korean welfare recipients and, thus, the exposure arguably reduced the likelihood of believing the misinformation.

However, in total, the number of searchers who relied on negative websites was larger than those who relied on positive websites (32.24% vs. 9.29%). This result suggests that the information available online is disproportionately negative against Zainichi Koreans. When available information is lopsidedly negative, online searchers are more likely to be exposed to negative rather than positive information at the initial stage of search even when they may check multiple websites by the end of the search. As negative affective response invoked by initial exposure to negative information leads to updating of online tallies, it is expected to have a persistent affective effect even after the misinformation is corrected, resulting in belief echo.

**S2 Fig** Contents of the websites found to be the most informative (Study 1).
