# Supplementary material for: Can “Googling” correct misbelief? Cognitive and affective consequences of online search
Source: PLoS One. 2021 Sep 22;16(9):e0256575. doi: 10.1371/journal.pone.0256575 (PMC8457483; doi:10.1371/journal.pone.0256575)
Supplement: S3 File — (DOCX) [file pone.0256575.s003.docx]

**S3 File. Covariate balance of Study 2**

|  | Control | Original treatment | Directional goal | Accuracy goal | Chi-squared test | ANOVA |
| --- | --- | --- | --- | --- | --- | --- |
| % Female | 52.36 | 49.82 | 52.01 | 54.33 | χ^2^(3) = 2.24, p = 0.53 |  |
| Age | 35.83 | 34.99 | 35.84 | 34.97 |  | *F*(3, 2185) = 1.46, *p* = 0.22 |
| Education (1–3) | 2.40 | 2.40 | 2.34 | 2.37 |  | *F*(3, 2185) = 0.62, *p* = 0.60 |
| % Party identity: LDP | 47.10 | 40.84 | 43.43 | 42.54 | χ^2^(3) = 4.69, p = 0.20 |  |
| % Party identity: DPJ | 5.43 | 6.41 | 6.75 | 6.45 | χ^2^(3) = 0.92, p = 0.82 |  |
| % Party identity: JRP | 5.07 | 5.13 | 5.66 | 5.16 | χ^2^(3) = 0.24, p = 0.97 |  |
| % Party identity: JCP | 3.80 | 4.95 | 5.11 | 5.52 | χ^2^(3) = 1.94, p = 0.59 |  |
