# Supplementary material for: Can “Googling” correct misbelief? Cognitive and affective consequences of online search
Source: PLoS One. 2021 Sep 22;16(9):e0256575. doi: 10.1371/journal.pone.0256575 (PMC8457483; doi:10.1371/journal.pone.0256575)
Supplement: S4 File — (DOCX) [file pone.0256575.s004.docx]

**S4 File. Content analysis of the most informative websites (Study 2)**

As in Study 1, we accessed and saved all the contents of websites reported by the participants as “the most informative website during the search.” Two trained coders independently coded the contents of those websites. There were 162 unique websites among the 1,608 responses. The coding indicated that 31.47% of the 1,608 responses were online news websites, followed by websites of traditional mass media (19.84%) and blogs (15.67%). Cohen’s Kappa was 0.64.

After excluding 55 unique websites that were not relevant to the issue of Zainichi Korean welfare recipients (148 responses), the coders categorized the remaining 1,460 responses into one of three types. 1: Websites that explicitly accuse Zainichi Koreans of illegitimately receiving welfare (32.67%; negative websites); 2: websites that explicitly defend Zainichi Koreans for legitimately receiving welfare (14.32%; positive websites); and 3: websites that are fact-based and neutral regarding the Zainichi Korean welfare recipients (53.01%; neutral websites). Cohen’s Kappa was 0.54. The three types of treatment were not associated with the type of “the most informative website during the search” (χ^2^(4) = 0.68, *p* = 0.95).

We first replicated the same content analysis as in Study 1. The results are presented in Figure OA2. In summary, confirmation bias is weak at best and most participants found fact-based, neutral websites to be the most informative (53.01%), which arguably led to the overall corrective effect of online search. At the same time, heavier reliance on the negative websites (32.67%) than on the positive websites (14.32%) could have promoted belief echo.

**S4 Fig1** Contents of the websites found to be the most informative (Study 2).

Next, by using the pretreatment subjective truthfulness of the statement from the three treatment groups, the *change* of subjective truthfulness before and after the search was operationalized. Among the three treatment groups (n = 1,608), 43.43% judged the misinformation to be false both before and after the search, 15.88% judged it to be false before but to be true after the search, 24.50% judged it to be true before but to be false after the search, and 16.19% judged it to be true both before and after the search. The change of subjective truthfulness or lack thereof is illustrated for each of the four types of “the most informative website during the search” in Figure OA3.

**S4 Fig2** Change of subjective truthfulness and the type of “the most informative website during the search.”

The results of neutral and positive websites are similar in the sense that the number of searchers who corrected their misbelief is larger than those who newly accepted the misbelief after the search, which indicates that relying on these types of websites tends to reduce the misbelief. In contrast, the number of searchers who newly accepted the misbelief after the search is the largest among those who found the negative websites to be the most informative, suggesting that online search can aggravate the misbelief.
